# Evaluation of the Ecological Environment Quality of the Kuye River Source Basin Using the Remote Sensing Ecological Index

**DOI:** 10.3390/ijerph191912500

**Published:** 2022-09-30

**Authors:** Qiang Liu, Feihong Yu, Xingmin Mu

**Affiliations:** 1 College of Resources and Environmental Engineering, Tianshui Normal University, Tianshui 741000, China; 2 State Key Laboratory of Soil Erosion and Dryland Farming on Loess Plateau, Institute of Soil and Water Conservation, Chinese Academy of Sciences and Ministry of Water Resources, Yangling 712100, China

**Keywords:** remote sensing ecological index, ecological quality, spatial auto-correlation, human activity stress, climate change

## Abstract

Landsat remote sensing images obtained from 2000, 2005, 2010, 2015, and 2020 were analyzed. The normalized vegetation index (NDVI), moisture index (WET), land surface temperature (LST), and normalized building-soil index (NDBSI) were extracted based on the four aspects of greenness, humidity, heat, and dryness. The Remote Sensing Ecological Index (RSEI) was calculated using principal component analysis to quantitatively analyze and dynamically monitor and evaluate the ecological environment changes in the Kuye River Basin over the past 20 years. From the perspective of spatial and temporal distribution, the ecological and environmental quality of Kuye River Basin had a downward trend from 2000 to 2020. The overall RSEI grade was medium or poor, and the average RSEI decreased. The proportion of excellent and good grade watershed areas decreased, whereas that of medium, low, and poor grade watershed areas increased over the study period. Spatially, RSEI decreased gradually from southeast to northwest. The degraded areas were mainly distributed in urban areas with frequent human activities. Conversely, the superior eco-environmental quality areas were mainly distributed in eastern sections of the watershed. Compared with 2000, the eco-environmental quality of the Yulin urban area and Shenmu County in the southern section of the watershed are worsening.

## 1. Introduction

The level of ecological environment quality is related to human production [1], economic and social activities, and interactions of the three factors which can restrict and affect the organic whole [2]. The ecological environment quality not only affects the quality of life of the human inhabitants, but also directly affects the stable sustainable development of the economy [3]. Ecological environmental quality assessment monitors the ecological environmental quality by investigating and analyzing ecological environmental quality of actual situations [4]. The process utilizes scientific experimental methods, data, evaluation indicators, and an evaluation system to analyze and assess changes in ecological environmental quality [5]. With the development of science and technology, human material civilizations have reached unprecedented heights but remain increasingly associated with serious ecological environment issues [6]. Humans excessively extract natural resources in the blind pursuit of their economic interests [7]. The contradiction between economic development and the allocation of resources is increasing and poses a serious threat to the survival and development of human beings [8]. Globally, people are paying increasing attention to the ecological environment. The concept of “sustainable development” was introduced at the United Nations Conference on Development and Environment in Brazil in 1992 [9]. It provided clear recognition of the need to protect the environment while pursuing economic development. Protecting the environment and achieving sustainable development are arduous tasks facing the entire world [10]. From the global perspective, China’s ecological and environmental problems are pressing. China’s economy is developing rapidly, but the process of development can excessively consume natural resources, leading to a series of ecological and environmental problems, such as land degradation, soil erosion, and air pollution among many others.

The watershed provides ecological services and the economic development functions of climate regulation, environmental purification [11], fresh water supply, navigable power generation, and an irreplaceable role in human production and life [12]. Simultaneously, the watershed is ecologically vulnerable [13]. When humans improperly use one or more functions of a watershed, it often has negative impacts on the ecological environment of the watershed [14], which impacts its ability to self-integrate into normal operations [15], resulting in ecological imbalance and environmental damage of the watershed [16]. Therefore, real-time, rapid, and accurate monitoring of the watershed ecological environment is an important link in watershed ecological protection. As one of the main tributaries of the Yellow River and one of the main sediment sources in the Yellow River, the Kuoye River is an important focus for soil erosion prevention and control in China. In this area, the contradiction between resources and ecological environment is prominent [17]. The Kuye River Basin is rich in coal resources, and massive coal mining has caused a series of problems in the ecological environment [18]. Mining has resulted in vegetation destruction, soil erosion, desertification, dust pollution, and other environmental problems. The surrounding environment of the coal mining area is harsh with significantly changed topography and geomorphology with reduced vegetation cover, aggravating soil erosion. Recently, the Chinese government began returning farmland to forest or grassland, which has improved the vegetation coverage of the Kuye River Basin [19]. However, the risk of environmental damage through coal mining remains. Therefore, a thorough understanding of the temporal and spatial variation of eco-environmental quality in Kuye River Basin provides a basis for environmental governance in the basin.

Remote sensing technology provides a large range of observations, fast acquisition speeds, large volumes of data, and accurate information [20]. It has great advantages in land and resource investigations and disaster monitoring [21]. Compared with traditional site-specific measurement methods, remote sensing technology can effectively save manpower and material resources. The development of remote sensing technology provides rich data for the dynamic monitoring of regional ecological environment quality [22]. Remote sensing technology provides timely results of changing characteristics in ecological environment quality, helping us coordinate the relationship between economy and environmental protection. It is an important technical support in our pursuit to achieve sustainable development. Remote sensing technology can aid monitoring of the quality of ecological environment. Remote sensing satellites can obtain the evaluation index of the earth’s surface and use geographic information technology to analyze the status of the ecological environment [23].

Remote Sensing Based Ecological Index (RSEI) provides new remote sensing technology [15]. RSEI integrates multiple indicators that intuitively reflect the Ecological environment and mainly based on natural factors. RSEI can objectively and quantitatively evaluate the regional ecological environment. Concurrently, the evolution of the ecological environment can be analyzed in time and space [24]. The RSEI coupled with the green index, humidity index, dryness index, and heat index can be combined to identify ecological problems by using principal component analysis to solve the rational weight settings and visualize ecological environmental quality [25]. Presently, RSEI evaluates the eco-environmental quality of cities and mining areas. Relatively few studies use this technique for the eco-environmental quality assessment of watershed systems. The factors influencing changes in watershed ecological environment are rarely discussed. We focus on the Kuye River Basin and selected the Landsat remote sensing images from 2000, 2005, 2010, 2015, and 2020 to extract the NDVI, WET, LST, and NDBSI based on the four aspects of greenness, humidity, heat, and dryness, respectively. RSEI provides the ecological environmental changes in the Kuye River Basin over the past 20 years using quantitative analysis with dynamic monitoring. Our research objective is to use remote sensing and GIS methods to extract ecological environment assessment indicators, to track and monitor the ecological environment, which can effectively grasp the ecological environment quality of the watershed, improve the quality of decision-making, and quickly prevent the excessive deterioration of the ecological environment. 

## 2. Materials and Methods

### 2.1. Study Area

**Figure 1:**
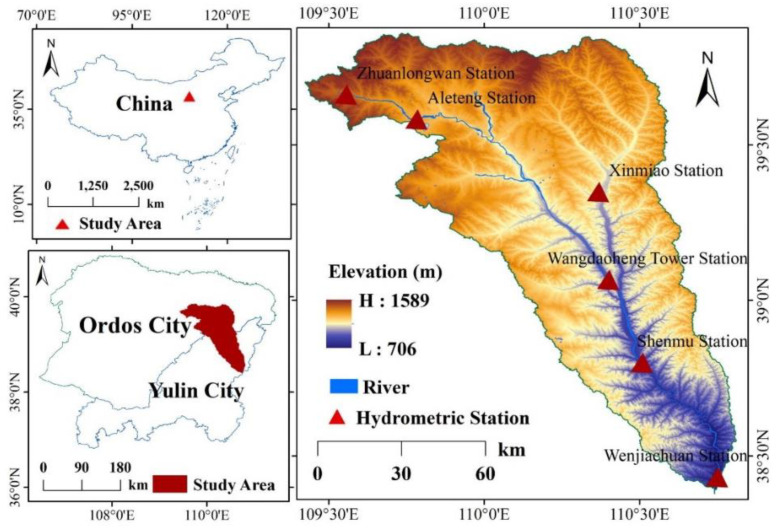
A map displaying the location of the Kuye River Basin.

The basin is located 38°28′–39°52′ north and 109°00′–110°52′ east. Kuye River is the second largest tributary of the middle reaches of the Yellow River. It originates from Badingou, Dongsheng City, Inner Mongolia Autonomous Region. The Kyye River has a total length of 242 km and a total basin area of 8706 km^2^. It flows through Ejin Horo Qi and Fugu, Shaanxi Province and into the Yellow River. The topography of the Kuye River Basin is complex and diverse. The terrain is high in the northwest and low in the southeast. The surface vegetation is scarce, the forest and grass coverage is low, the underlying surface conditions are complex, and there are desert steppe, meadow, artificial irrigation, and agricultural vegetation. The climate type of the basin is semi-arid continental monsoon in the north temperate zone. The annual average precipitation is 419 mm, which varies greatly over a year. The annual maximum precipitation mainly occurs in July and August, which accounts for 55–65% of the total annual precipitation. The rainfall is spatially distributed in the southeast and the northwest. The annual average temperature is 8.2 °C, with the highest temperatures in July (Figure 1).

### 2.2. Date Source and Date Processing

In 2021, the United States geological survey (USGS) used the GEE platform (https://developers.google.com/earth-engine/datasets/catalog/landsat, accessed on 27 September 2022) to create a new Landsat Collection Level 2 dataset containing positively-corrected Landsat surface reflectance and surface temperature bands [26]. In our study area, the bands better reflect the ecological environment conditions in the vegetation growing season. We selected images from the dataset with cloud cover less than 20% in the years of 2000, 2005, 2010, 2015, and 2020. We used the cloud removal algorithm provided in GEE platform to remove the cloud and its shadow using QA_PIXEL of the pixel quality band and synthesized the multi-layer image into a multi-band image with good quality using the median value. To ensure sufficient image quantity and quality, we selected only images with the cloud cover below 20%. Due to the large time scale, the data sets used different satellites/sensors in different years. Landsat 7 ETM images provided the data for 2000 and 2005, Landsat 5 TM images were used for the 2010 data, and Landsat 8 Oli/TIRS images were used for the 2015 and 2020 data.

The runoff and sediment transport data from the Kuye River Basin from 1956–2009 were obtained from the Yellow River Hydrological Yearbook, while the same data for 2010–2020 were obtained from the Yellow River sediment Bulletin. The precipitation data for 1956–2020 were obtained from the surface daily meteorological observation data of four meteorological stations (Dongsheng, Hequ, Yulin, and Xingxian) from the China Meteorological Data Network (http://data.cma.cn, accessed on 27 September 2022). The spatial interpolation used the mean values for the basin to analyze the climate change trends.

### 2.3. Methodology 

#### 2.3.1. RSEI Calculation

The functions of principal component transform of remote sensing image are image compression, image denoising, image enhancement, image fusion, and feature extraction. Principal component analysis (PCA) is used to reduce the dimension of the index bands of greenness, humidity, heat, and dryness to obtain the irrelevant linear component data, and then extract the components with large contribution rate and discard the redundant component data with little information and analyze the component data with large weight. In our study, the dimension of the remote sensing data is degraded by compressing the component data of the four indicators, removing redundant data, enhancing the remote sensing image, and obtaining the eigenvalues of the component indicators after principal component analysis. 

The RSEI contains four indices—greenness, wetness, dryness, and heat. These are represented by the NDVI, WET, NDBSI, and LST, respectively [27]. They are obtained by remote sensing and then coupled in a PCA. The greenness index (NDVI) was calculated using the NDVI. NDVI is an important biophysical parameter reflecting the state of surface vegetation, which is closely related to plant biomass, vegetation coverage, and leaf area index. In the field of ecological environment, due to the expansion of ecosystem research results at the landscape patch level, NDVI has become the connection point of spatial scale expansion. Vegetation Index can be used to reflect the change in vegetation cover and land use, to realize the study of ecological environment quality. Vegetation connects soil, atmosphere, and water in the terrestrial ecosystem, regulates the material and energy exchange between the interior and exterior of the ecosystem, and has an important influence on the global energy cycle and the biochemical ring of substances. The vegetation has obvious interannual and seasonal changes. When the vegetation cover changes in the region, it will have a direct impact on the ecological environment quality of the whole region. The wetness index (WET) was calculated using a tasseled cap transformation of the remote sensing images. The dryness (NDBSI) was obtained using the bare land index and the built-up area index as average values. The heat index (LST) was obtained by calculating the actual temperature of the ground surface using thermal infrared. The RSEI is defined as follows:

(1) $$\mathrm{RSEI}=f\left(\mathrm{NDVI},\;\mathrm{WET},\;\mathrm{NDBSI},\;\mathrm{LST}\right)$$

① Greenness

(2) $$\mathrm{NDVI}=\left(\mathrm{NIR}-R\right)/\left(\mathrm{NIR}+R\right)$$

where NIR and R are the reflectance of the near -infrared and red bands, respectively.

② Wetness

The tasseled cap transformation algorithm can compress and remove redundant data. Its greenness, humidity, and brightness components are directly related to the physical parameters of the surface [25], and widely used in ecological environment monitoring. The moisture component of the Tasseled Cap Transformation Algorithm is closely related to the soil moisture and vegetation. In this study, the moisture component of the silk hat transformation provides the humidity index to reflect the surface humidity in the basin. When calculating the humidity component of the silk hat transformation, the coefficients of each band of the different Landsat sensor images were different, which are expressed as follows:

(3) $${\;\mathrm{WET}}_{\mathrm{TM}}=0.0315\rho 1+0.02021\rho 2+0.3102\rho 3+0.1594\rho 4-0.6806\rho 5-0.6109\rho 6$$

(4) $${\mathrm{WET}}_{\mathrm{ETM}}=0.2626\rho 1+0.2141\rho 2+0.0926\rho 3+0.0656\rho 4-0.7629\rho 5-0.5388\rho 6$$

(5) $${\mathrm{WET}}_{\mathrm{OLI}}=0.1511\rho 1+0.1973\rho 2+0.3283\rho 3+0.3407\rho 4-0.7117\rho 5-0.4559\rho 6$$

where Wet (TM), wet (ETM+) and wet (Oli) are the moisture components of the Tasseled Cap Transformation Algorithm of the Landsat TM, ETM+, and Oli images, respectively. *ρ*_blue_, *ρ*_green_, *ρ*_red_, *ρ*_NIR_, *ρ*_SWIR1_*,* and *ρ*_SWIR2_ are the surface reflectance of Landsat TM, ETM+, and Oli images in blue, green, red, near infrared, short wave infrared 1, and short-wave infrared 2 bands, respectively.

③ Dryness

Soil drying causes serious pollution in the regional ecological environment. The dryer the soil, the more harm to the environment. In addition to bare soil (such as rock, sandy land, bare ground), urban building areas also cause soil “drying” [28]. Therefore, the dryness index (NBDSI) was developed using the bare soil index (IS) and building index (IBI), which can be calculated using the following formula [29]:

(6) $$\mathrm{SI}= [\left(\rho_{\mathrm{swirl}}+\rho_{\mathrm{red}}\right)-\left(\rho_{\mathrm{nir}}+\rho_{\mathrm{blue}}\right)]/ [\left(\rho_{\mathrm{swirl}}+\rho_{\mathrm{red}}\right)+\left(\rho_{\mathrm{nir}}+\rho_{\mathrm{blue}}\right)]$$

(7) $$\mathrm{IBI}=\frac{2\rho_{\mathrm{swirl}}/\rho_{\mathrm{swirl}}+\rho_{\mathrm{nir}}- [\rho_{\mathrm{nir}}/\left(\rho_{\mathrm{nir}}+\rho_{\mathrm{red}}\right)+\rho_{\mathrm{green}}/\rho_{\mathrm{green}}+\rho_{\mathrm{swirl}}]}{2\rho_{\mathrm{swirl}}/\rho_{\mathrm{swirl}}+\rho_{\mathrm{nir}}+ [\rho_{\mathrm{nir}}/\left(\rho_{\mathrm{nir}}+\rho_{\mathrm{red}}\right)+\rho_{\mathrm{green}}/\rho_{\mathrm{green}}+\rho_{\mathrm{swirl}}]}$$

(8) $$\mathrm{NDBSI}=\left(\mathrm{SI}+\mathrm{IBI}\right)/2$$

where Landsat TM, ETM+, and Oli correspond to the reflectance in each band. SI is the bare earth index, and IBI is the building index.

④ Heat (LST)

The atmospheric correction method was used for the inversion of LST [30]. After selecting the thermal infrared band of the image for the radiometric calibration, the blackbody radiation brightness was obtained [31], and the LST was calculated using the Planck function [11], with the following formula [32]:

(9) $$\;\mathrm{LST}=K_{2}/\ln[\frac{K_{1}}{B\left(\mathrm{LST}\right)}+1]$$

(10) $$B\left(\mathrm{LST}\right)= [L_{\lambda }-L↓-\tau \left(1-\varepsilon \right)L↑]/\tau \varepsilon$$

(11) $$L_{\lambda }= [\varepsilon B\left(\mathrm{LST}\right)+\left(1-\varepsilon \right)L↑]\tau +L↓$$

where L_λ_ is the brightness value of the thermal infrared radiation; B (LST) is blackbody radiation brightness; LST is the real surface temperature; Epsilon for Surface emissivity is τ; L↓ and L↑ are the atmospheric profile parameters.

Since the four indicators have their own dimensions and each indicator has an unbalanced weight, there was no way to directly collate them into the principal component analysis. Therefore, the data was normalized into [0, 1] prior to performing the PCA. The following formula for each indicator was as follows [33]:

(12) $${\mathrm{NI}}_{i}=\left(I_{i}-I_{\min}\right)/\left(I_{\max}-I_{\min}\right)$$

(13) $$\mathrm{RSEI}=\mathrm{PCA} [f\left(\mathrm{NDVI},\;\mathrm{WET},\mathrm{NDBSI},\;\mathrm{LST}\right)]$$

(14) $$\mathrm{RSEI}=\left({\mathrm{RSEI}}_{0}-{\mathrm{RSEI}}_{0}{}_{\min}\right)/\left({\mathrm{RSEI}}_{0}{}_{\max}-{\mathrm{RSEI}}_{0}{}_{\min}\right)$$

where NI_i_ is the result of the image standardization; I_i_ is the numerical pixel value of the image. I_max_ and I_min_ are the maximum and minimum values of the image pixel, respectively; PCA is the principal component analysis, and f is the set of functions used to perform the PCA; The maximum and minimum values of RSEI_0_ are expressed as RSEI_0max_ and RSEI_0min_, respectively.

#### 2.3.2. Spatial Autocorrelation Analysis 

Spatial autocorrelation is an important index to investigate whether the attribute value of an element significantly correlates with the attribute value of its adjacent space. This reveals the correlation of the attribute eigenvalues between spatial reference units and their adjacent spatial units. We used both Moran’s index (global spatial autocorrelation) and the local spatial correlation index (Lisa) to analyze the spatial correlation of the eco-environmental quality. The global spatial autocorrelation analysis investigated the overall spatial distribution characteristics of the regional RSEI which was represented by Global Moran’s I using the following formulas [34]:

(15) $$\mathrm{Global}\;\mathrm{moran\prime s}=\frac{\sum_{i=1}^{n}\sum_{j=1}^{m}\omega_{\mathrm{ij}}\left(x_{i}-\bar{X}\right)\left(x_{j}-\bar{X}\right)}{S^{2}\sum_{i=1}^{n}\sum_{j=1}^{m}W_{\mathrm{ij}}}$$

where x_i_ is the attribute value of position i; N is the total number of grids in the study area; W_ij_ is the weight of the matrix, which represents the relationship of the spatial objects, i = 1, 2, 3..., N, j = 1, 2, 3..., M, W_ij_ = 0 when i and j are adjacent. The Moran’s I ranges from approximately +1 (positive spatial autocorrelation) to −1 (negative autocorrelation), with zero indicating no spatial autocorrelation.

LISA can reflect the local correlation of the spatial distribution of an object’s attributes. Even when I in the global Moran’s I is 0, there may be local clustering. Therefore, it is necessary to combine the LISA and Global Moran’s I analyses. The formula was expressed as follows:

(16) $$\mathrm{LISA}=\left(\frac{\left(x_{i}-\bar{X}\right)}{m}\right)\sum_{j=1}^{m}W_{\mathrm{ij}}\left(x_{i}-\bar{X}\right)$$

(17) $$m=\frac{\left(\sum_{j-1,\;}^{m}j\neq 1x_{i}^{2}\right)}{\left(n-1\right)}-x^{2}$$

where LISA represents the spatial clustering of similar values (high value or low value) around the spatial unit, and a negative LISA represents the spatial clustering between dissimilar values.

The results of the local spatial autocorrelation analysis generated a LISA cluster, which presented five spatial distribution types: high-high (H-H) cluster, low-low (L-L) cluster, high-low (H-L) outliers, low-high (L-H) outliers and insignificant values. H-H was the presence of a high value surrounded by a high value, and L-L was the presence of a low value surrounded by a low value. H-L indicates a high-value anomaly and L-H indicates a low-value anomaly. Non-significant values indicate the attribute values are close to randomly distributed.

#### 2.3.3. RSEI Flow Chart

**Figure 2:**
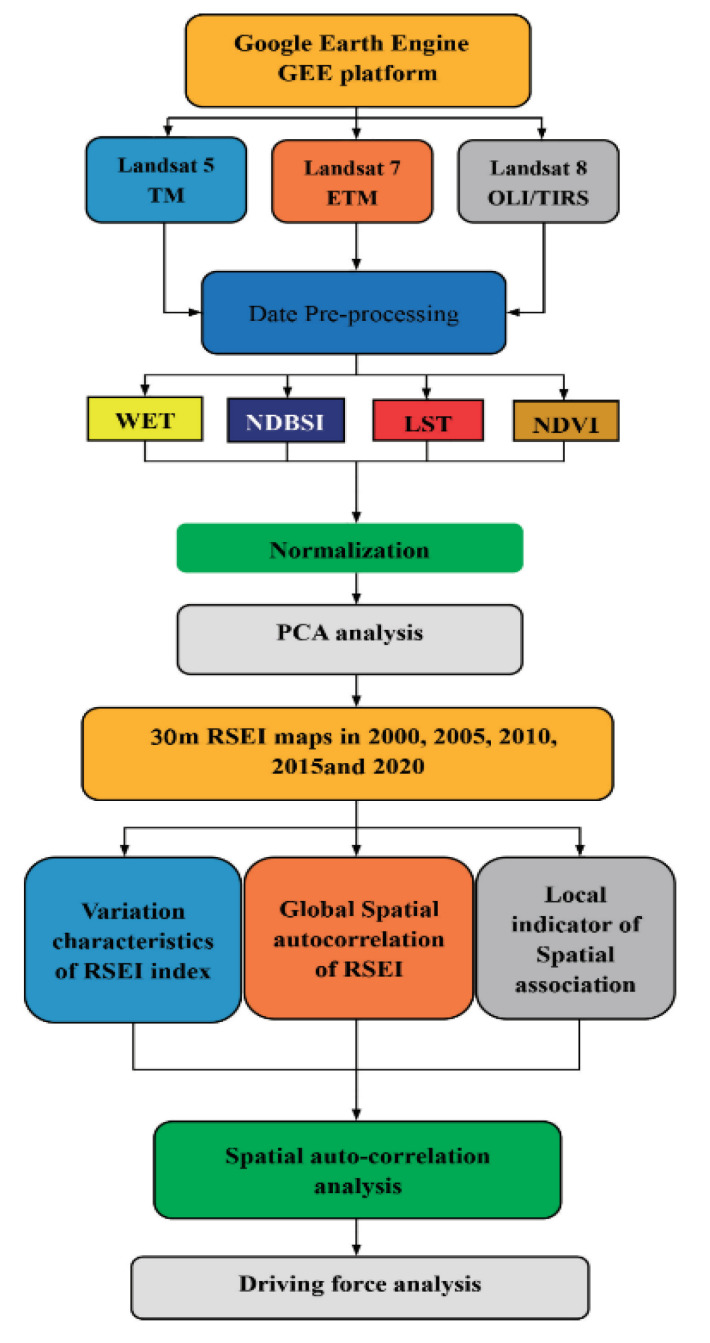
Workflow chart demonstrating the ecological quality in the Kuye River.

RSEI uses a principal component transformation by aggregating the main information provided by the NDVI, WET, NDBSI, and LST indicators into the first principal component so that they can be described using a single indicator [35]. This method integrates the information closely related to the four indicators and has advantages in constructing a RSEI. The AM, ETM, and image data stored in the GEE cloud platform were used in the spatiotemporal distribution maps of the RSEI in Kuye River Basin in 2001, 2005, 2010, 2015, and 2020 and generated using the PCA. The Moran’s index (global spatial auto-correlation) and LISA (local indicator of spatial association) analyzed the spatial correlations for the eco-environmental quality. The driving factors of the eco-environmental quality changes were analyzed [17]. The lower the RSEI value, the worse the ecological conditions. The RSEI was further normalized between 0 and 1, in which 0 indicated an extremely poor status and 1 indicated an excellent status. Then, following previous studies, the normalized RSEI was reclassified into five levels (excellent: 0.8—1.0; good: 0.6—0.8; moderate: 0.4—0.6; fair: 0.2—0.4; poor: 0—0.2) using an equal interval of 0.2. A detailed workflow of this study is provided in Figure 2.

## 3. Results

### 3.1. PCA of RESI

Table 1 states the contribution rate of the eigenvalue of the first principal component of the RSEI in the fifth period is above 85%, and the contribution rate of each index has the same positive and negative distribution in the first principal component. Specifically, the contribution rate of NDVI (representing vegetation coverage) and WET (representing environmental humidity) in the first principal component is positive. LST (representing land surface temperature) and NDBSI (representing hardening degree of buildings and bare soil) were negative values. This result indicates NDVI and WET played a promoting role in improving the ecological environment. Conversely, LST and NDBSI played a restraining role in improving the ecological environment. This result was consistent with the reality of the effects of the four indicators on the ecological environment. In 2000, 2005, 2010, 2015, and 2020, the contribution rate of the first principal component (PC1) was 85.61, 86.17, 86.83, 85.95 and 90.01%, respectively. Therefore, all are greater than 85%, indicating that the first principal component was based mainly on the information of the four indicators. Moreover, the contribution of the four component indicators in the PC1 is relatively stable. Therefore, PC1 replaced the four component indicators in creating the RSEI.

In 2000, 2005, 2010, 2015, and 2020, the contribution of the dryness index in PC1 was −0.851, −0.853, −0.861, −0.879, and −0.891, respectively. This demonstrates a gradually increasing trend. The cause is the urbanization of Kuye River Basin which accelerated after 2000, increasing the area of build-up land. The dryness index reflects the change in the construction land in the study area. This phenomenon demonstrates that the urban planning and construction of the Kuye River Basin has obvious influences on the urban eco-environmental quality (especially the increased construction land area caused by urban expansion) which has become the key factor affecting the eco-environmental quality of the Kuye River Basin.

### 3.2. Variation Characteristics of RSEI Index

#### 3.2.1. NDVI

**Figure 3:**
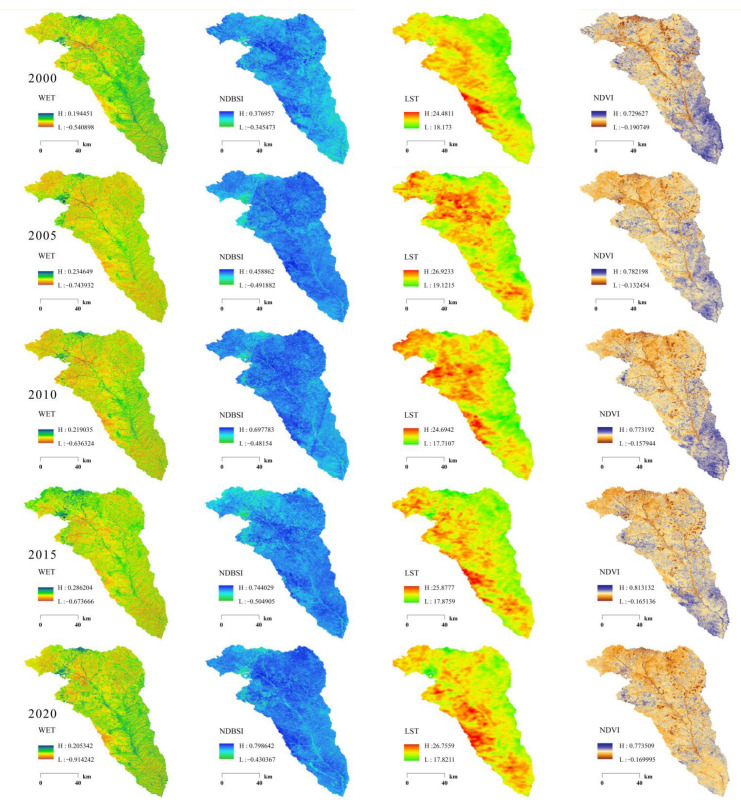
Temporal and spatial distribution of various indicator results in Kuye River Basin.

The higher the greenness value, the higher the vegetation coverage rate and the better the ecological environment quality [1]. Conversely, the lower the greenness value, the lower the vegetation coverage and the worse the ecological environment quality [17,27]. The greenness index in 2000, 2005, 2010, 2015, and 2020 after the standardization treatment are between 0 and 1. As demonstrated in Figure 3, the overall distribution characteristics of the NDVI in the five different years are basically the same, with the characteristics of low in the northwest and high in the southeast, and the NDVI changes significantly in the southeast. In 2000, the whole Kuye River Basin was dominated by a high value area (blue), the NDVI high value area was mainly distributed in the south, and the NDVI low value area was mainly distributed in the north. In 2005, when compared with 2000, the low value range increased significantly (light brown), indicating that the vegetation coverage of the region was decreasing. In 2010, when compared with 2005, the NDVI value in northern China decreased slightly. In 2015, when compared with 2010, the NDVI value in southern China decreased slightly. In 2020, the NDVI value decreased significantly in the south when compared to 2015.

#### 3.2.2. WET

The higher the humidity value, the higher the surface water content, the better the ecological environment quality, and vice versa [31]. The humidity index in 2000, 2005, 2010, 2015, and 2020 after the standardization treatment is between 0 and 1. As demonstrated in Table 2, from 2000–2020, the humidity in Kuye River Basin initially decreased before increasing, but the overall trend was decreasing. This indicates the increase in vegetation coverage increased the demand for water, resulting in a decrease in the ground humidity. The mean values of the ground humidity index in 2000, 2005, 2010, 2015, and 2020 were 0.106, 0.014, 0.021, 0.036, and 0.051, respectively. As demonstrated in Figure 3, in 2000, the humidity in the eastern section of the basin and near the river were high, while the humidity in the northern section of the basin was low. In 2005, the WET value decreased significantly when compared with 2000. In 2010, there was a small increase in the WET value in the middle of the basin. In 2015, there was a small increase in the WET value in the north when compared to 2010. In 2020, there was a small increase in the WET value in the southern section of the watershed when compared to 2015. 

#### 3.2.3. NDBSI

The higher the NDBSI value, the more serious the soil degradation in the region and the worse the ecological environment quality. Conversely, a low NDBSI value relates to a high ecological quality [36]. The values of the dryness index in 2000, 2005, 2010, 2015, and 2020 after the standardization treatment is between 0 and 1. As demonstrated in Table 2, the mean values of the NDBSI index of the five stages were 0.282, 0.321, 0.332, 0.423, and 0.528, respectively. Therefore, as land construction in the Kuye River Basin increased the soil deteriorated sharply from 2000 to 2020 as economic development and urbanization accelerated. In addition, high intensity groundwater extraction led to decreases in surface water in the Kuye River Basin, an increase in the dryness index in some areas, and the deterioration of the ecological environment quality. 

#### 3.2.4. LST

In Figure 3, green and red represent areas with high and low heat values, respectively. The higher the heat value, the worse the ecological quality within the region. Table 2 shows the regions with high LST values in Kuye River Basin exhibited an upward trend from 2000 to 2020. The LST mean values in the five periods were 19.521, 20.139, 20.333, 20.536, and 20.602, respectively. Figure 3 graphically demonstrates the five-stage LST distribution map, the high temperature area (in red) encompasses the main urban area and areas of exposed soil, while the non-urban areas and the river possess an appropriate temperature (in green).

### 3.3. Spatiotemporal Characteristics of the RSEI Evolution in the Kuye River Basin

**Figure 4:**
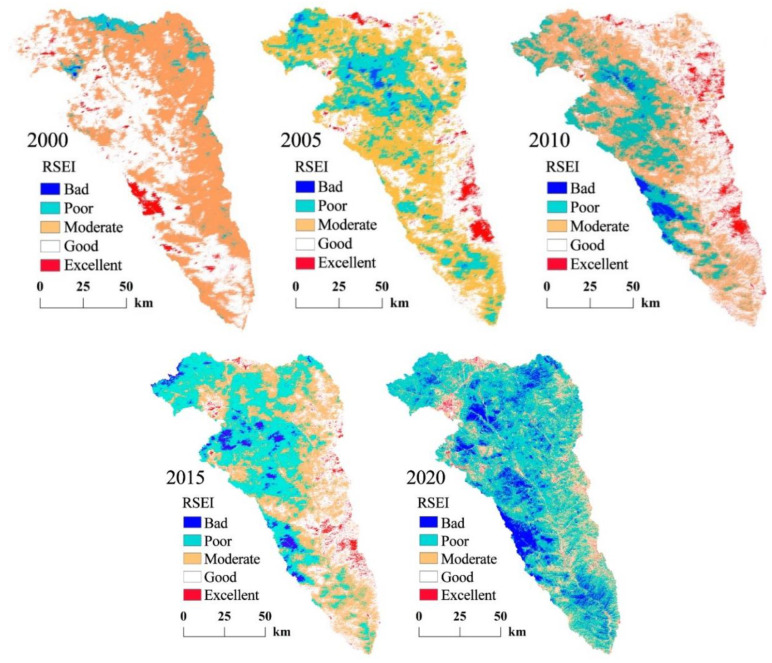
Spatial distribution of different RSEI quality levels in the Kuye River Basin in 2000, 2005, 2010, 2015, and 2020.

**Figure 5:**
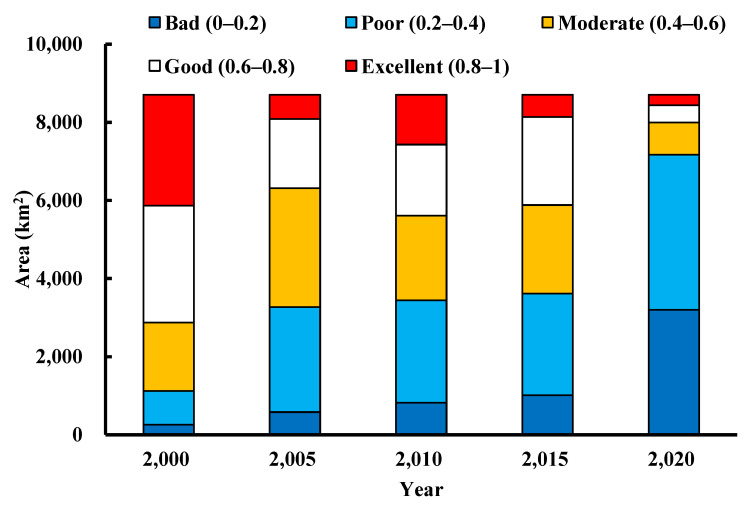
Area distribution of the ecological environment quality levels in the Kuye River Basin in 2000, 2005, 2010, 2015, and 2020.

Figure 4 provides the spatial and temporal distribution map of the ecological environmental quality in Kuye River Basin from 2000–2020. Red, white, yellow, light blue, and dark blue represent excellent, good, medium, poor, and bad ecological grades, respectively. The area (Figure 5) and proportion (Table 3) of each ecological level were calculated using the five RSEI maps from 2000, 2005, 2010, 2015, and 2020. As demonstrated in Figure 5, the area of moderate and poor RSEI increased over time. Conversely, the area of good and excellent RSEI decreased, indicating that the overall ecological environment quality of the Kuye River Basin was declining. Table 3 shows the sum of the proportions of the poor, level results (BPM%), and the sum of the proportions of good and excellent level (GE%) results. In 2000, 2005, 2010, 2015, and 2020, the results were 33.02%/66.98%, 72.51%/27.49%, 64.45%/35.55%, 67.56%/32.44%, and 91.80%/8.2%, respectively. The BPM% initially increased, then decreased and then increased again. Concurrently, GE% had the opposite pattern of initially decreased, then increased and then decreased again.

From 2000–2005, the area with high ecological and environmental quality in Kuye River Basin decreased significantly, from 66.98% (GE) to 27.49%. Firstly, the local climate conditions caused large areas of vegetation to die, resulting in decreased vegetation coverage. Second, the rapid economic development, population density, and per capita GDP growth increased the human activities, damaging the surface ecological environment. In 2005–2010, the area with high river basin ecological environment quality increased, this was due to the development of soil and water conservation engineering. The active promotion of the ecological construction engineering by river basin ecological environment construction, organization of local people in controlling desertification, development of agriculture in arid areas, solving the problems of food and clothing for the farmers, and adjusting the rural industrial structure. Ecological awareness drives the adjustment and optimization of land use structure, develops the river basin economy, and attempts to promote improvements in the ecological environment quality. From 2015–2020, the areas with high ecological and environmental quality in the Kuye River Basin decreased significantly. During this period, the rapid urbanization of the Kuye River Basin led to the deterioration of the ecological environment quality. The environment quality was especially impacted by the rapid urban population growth which involved the reclamation of new land, destroying the original soil structure. Urbanization affects the soil quality such as surface runoff, soil water infiltration, and soil water content, resulting in the reduction of surface vegetation coverage and potentially causing excessive soil and water loss. There are many coal mining enterprises in Kuye River Basin. Coal mining requires a lot of resources and energy, and discharges pollution. Coal mining lowers the underground water level, which reduces or ceases river water flow. The natural ecosystem of the Kuye River Basin is fragile, the cultivated land in the basin has sharply reduced. Land desertification is becoming increasingly serious, the forest resources have sharply reduced and are now limited. The freshwater resources are seriously limited, and the biodiversity is constantly decreasing. Therefore, the natural environment in sections of the basin have been seriously damaged, and the ecological environment quality has been significantly decreased.

Figure 4 demonstrates that in 2000 the RSEI level increased gradually from upstream to downstream and reduced gradually from east to west. The ecological environment quality is better downstream and is superior in the west. The precipitation diminishes from southeast to northwest. The temperature increased from east to west. The vegetation coverage was better in the lower reaches than in the upper reaches. From 2005 to 2015, excellent eco-environmental quality areas were mainly distributed in the eastern section of the watershed. Conversely, the eco-environmental quality in the Yulin urban area and Shenmu County in the southern section of the watershed showed a worsening trend when compared to the results in 2000. This change is mainly because coal mining in these regions caused serious environmental pollution and damage to the watershed. In 2020, the area of low ecological and environmental quality reached 91.8% (BPM). The low-quality areas were mainly distributed in the northwestern region of Kuye River Basin, which is the desert area of the southern edge of the Mu Us desert and covered by yellow sand. In the west, sandstorms cause serious erosion, and the area belongs to an ecologically fragile district. The RSEI level of this area is poor. The regional natural ecological environment conditions are relatively unsatisfactory with an unreasonable ecological structure, ecological environment destruction, a weak ecological function and resilience system, and a very difficult area for undertaking ecological restoration.

### 3.4. Spatial Auto-Autocorrelation Analysis of RSEI

**Figure 6:**
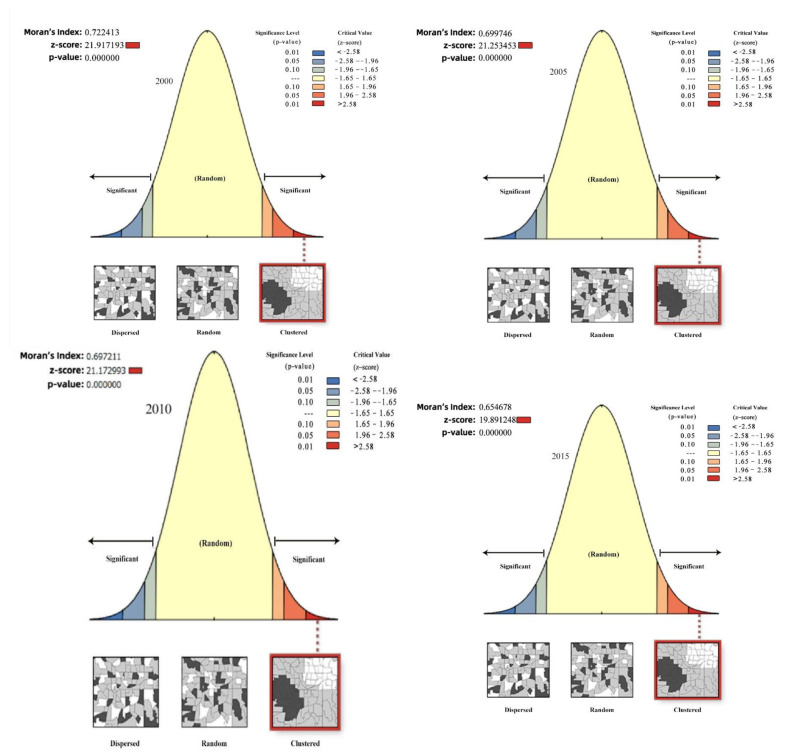

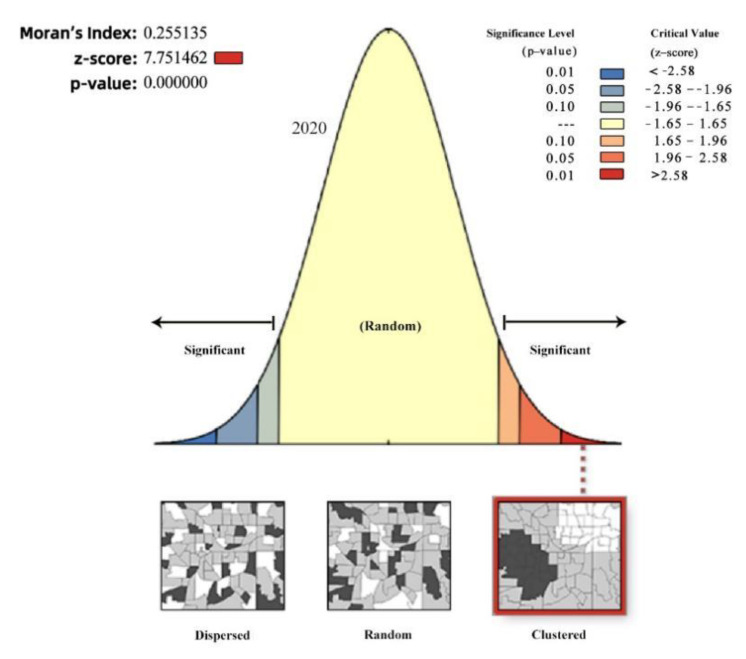
Moran’s map of the ecological environment quality levels in Kuye River Basin in 2000, 2005, 2010, 2015, and 2020.

Figure 6 provides the global Moran’s index and its corresponding Z values, which were 0.722 and 21.91 in 2000, 0.699 and 21.25 in 2005, 0.697 and 21.17 in 2010, respectively. In 2015, they were 0.654, 19.891, and in 2020, they were 0.255, 7.75, respectively, with higher Z values than the critical level of 1.96. At the significance level of 5%, the analysis revealed the RSEI was not a completely random state, with a significant correlation in space. RSEI positively correlated with spatial information. The remote sensing ecological index increases in areas with large spatial distributions of ecological environmental quality. Conversely, the remote sensing ecological index decreases if the spatial distribution of ecological environmental quality is small. From 2000 to 2020, Moran’s index had a downward trend, the degree of spatial autocorrelation became weaker, and the spatial distribution dispersed. This result was consistent with the spatial and temporal variation characteristics of the ecological environment quality level results.

### 3.5. Local Indicators of Spatial Autocorrelation of RSEI

**Figure 7:**
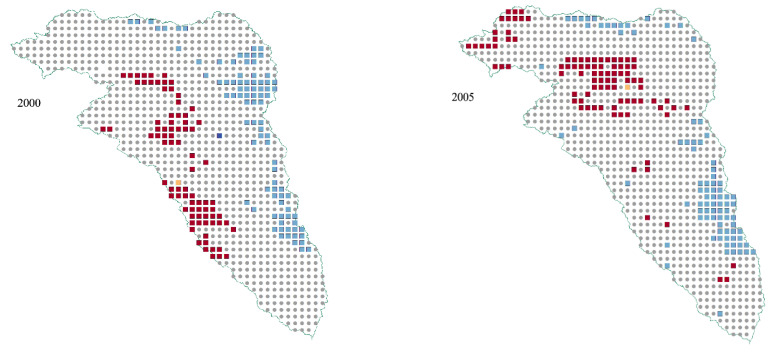

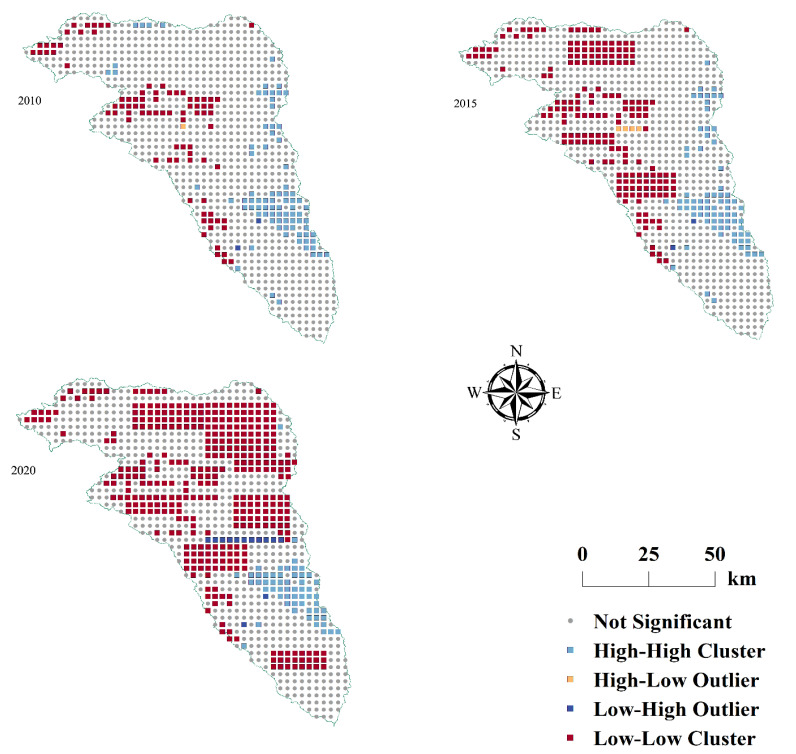
LISA map of the ecological environment quality levels in Kuye River Basin in 2000, 2005, 2010, 2015, and 2020.

To understand the spatial and temporal distribution of ecological environmental quality, we analyzed the local spatial correlation patterns of RSEI using LISA clustering. The LISA clustering diagram (Figure 7) revealed H-H clustering areas were mainly distributed in the east of the watershed. L-L was mainly distributed in the northwest of the watershed, as well as along the river and coal mining development zones. These areas are densely populated, especially the L-L cluster area which increased significantly from 2015 to 2020, due to the rapid urban growth and the increase in industrial and mining land. This development destroys the original soil structure, reduces the surface vegetation coverage, and potentially increases soil and water loss. Our results support previous studies. The area of built-up land in Kuye River Basin increased from 78.29 hm^2^ in 1980 to 877.25 hm^2^ in 2020. The built-up area has the highest growth rate among all the land use types, indicating that human activities rapidly expanded causing built-up land and generated a higher demand on the environment.

## 4. Discussion

### 4.1. The Influence of Natural Factors on the Ecological Environment

Good research results can indeed be obtained by using a variety of remote sensing data for ecological environmental quality assessment, but the time scale of these data is not long, so it is impossible to study the ecological environmental quality change of Kuye River Basin in 2000. Compared with other satellites, Landsat series satellite image data have large relative data storage, long time series and relatively moderate 30 m resolution. More importantly, GEE (Google Earth Engine) platform has level-2 remote sensing data synthesized by TOA of Landsat series released by USGS. Cloud interference and noise can be effectively removed, and seamless image stitching can be achieved to reduce the lack of image information. 

**Figure 8:**
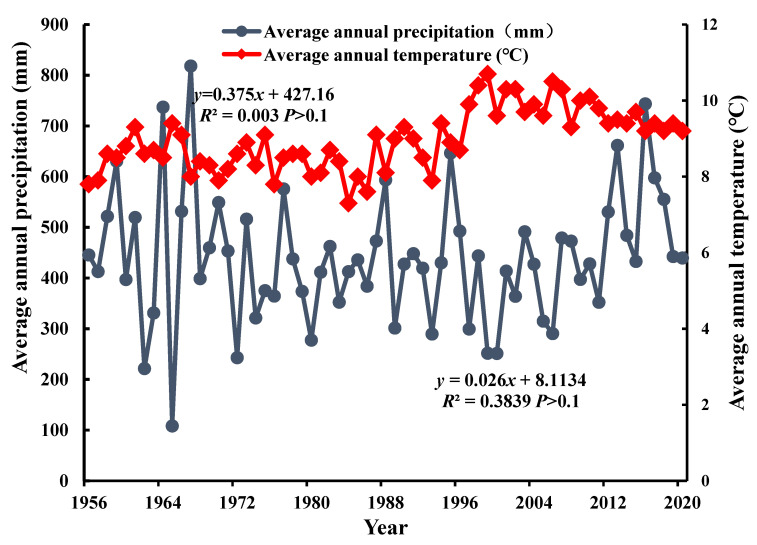
Variations in annual rainfall and temperature in Kuye River Basin from 1956 to 2020.

Greenness, humidity, heat, and dryness are important components of an ecological environment. The ecological index is based on the main information from the four indexes and better reflects the ecological environment quality of Kuye River Basin [37]. Each index of the ecological environment interacts and influences each other. When the temperature rises, the activity of some microorganisms increases, and the activity of some microorganisms decreases which changes the types and quantities of microorganisms, resulting in changes in soil oxygen content and fertility, RSEI index, and finally ecological environment quality. When rainfall increases, there is sufficient water in the basin, river runoff increases, plants grow, soil moisture content is rich, and soil structure is stable. Meanwhile, increasing rainfall causes soil erosion to intensify. There are serious impacts of soil erosion such as, soil internal structure changes, and finally RSEI is affected. Figure 8 demonstrates the annual precipitation change rate in the Kuye River Basin was 0.375 mm/A (*p* > 0.1). The average annual precipitation had an increasing trend that was not statistically significant, and the average annual temperature increased at a rate of 0.026 ℃/a in Kuye River Basin (*p* > 0.1). The precipitation changes did not differ statistically. However, the average annual temperature showed a statistically insignificant but increasing trend. Water and heat are two of the most important natural conditions for promoting vegetation growth and vegetation cover, which affect the ecological processes of plant communities and the plant growth cycle. Precipitation changes are directly related to NDVI and WET in this region, they can improve the quality of the local ecological environment [14]. Therefore, water and heat conditions play an important role in the ecological environment of the Kuye River. However, in the study area, both temperature and rainfall showed no significant increasing trend, while NDVI and WET showed a decreasing trend, indicating other factors are affecting NDVI and WET. NDBSI (which is negatively correlated with the ecological environment) contributes the most to RSEI, which corresponds to the climate warming characteristics in the Kuye River Basin as global climate change progresses. The negative correlation between LST and RSEI corresponds with the obvious heat island effect in the basin.

### 4.2. The Influence of Human Activities on Ecological Environment Change

Human activity refers to the various livelihood activities [38]. From 2000 to 2020, the population density in Kuye River Basin showed a straight upward trend, with the population increasing from 500,000 in 2000 to 1 million in 2020, and the per capita GDP had a “J-shaped” growth trend. However, the basin area is fixed, and the ecological carrying capacity is limited, so the population and economic growth have gradually approached the environmental limit. Population growth not only causes a higher consumption of resources, but also enlarges the ecological disturbance. The increased population leads to frequent human activities. To meet human demands, it is necessary to accelerate the exploitation of environmental resources, forcing the city to continuously expand outward, changing other land types into construction land, and ultimately changing the ratio of the land type structure, affecting the ecological environment quality.

**Figure 9:**
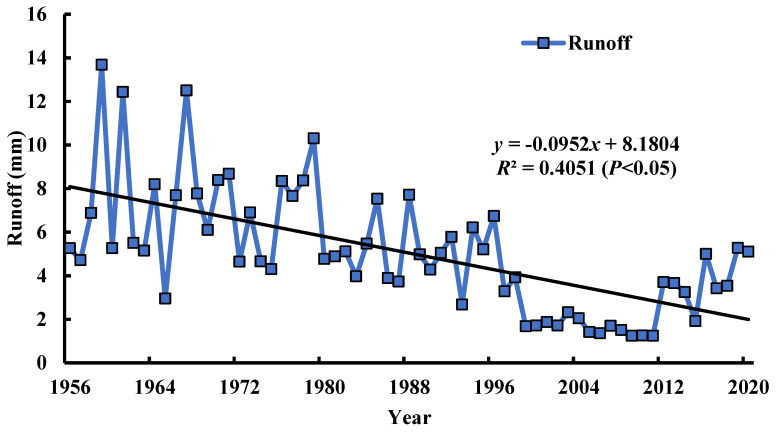
Change trending chart of the annual runoff in the Kuye River Basin from 1956 to 2020.

Population growth and economic development requires human beings to consume resources for faster development, which leads to vegetation destruction, changes in land use type, and the decline of the ecological environment quality. The Kuye rivers region is mainly in the arid areas which were inundated for a long time. As the population increased in the basin, the river was used for irrigation and the groundwater was continuously exploited. As a result, the river surface runoff is significantly reduced (Figure 9), and a reduced surface runoff can lead to a reduced water vapor content, reducing the soil moisture. In the 1980s, a large coal reserve was discovered in the Kuye River Basin, accounting for 20% of China’s coal reserves. Local people began to exploit the coal, and the volume of mined coal is continuously increasing. The average annual volume of mined coal increased from approximately 1 × 107 t in 1997 to 2.5 × 108 t in 2017. The underlying surface conditions have changed, affecting the discharge of surface runoff. In addition, most coal mining enterprises are located in mountainous areas, and coal mining seriously damages the surface vegetation, resulting in a significant decrease in vegetation coverage, a significant reduction in river runoff, and often interrupts water flow. The large-scale afforestation in the Kuye River Basin also negatively impacts the ecological environment. One of the main manifestations of this is the likelihood of increased drought events in dry years. Kuye river basin is attempting to return the farmland to forest which should dramatically change the watershed vegetation quantity, increasing the frequency and intensity of drought events. An increased vegetation coverage may result in a decline in the soil moisture over a long time scale. This will bring adverse effects on the returning farmland to forest References project, causing dwarf vegetation with stunted growth and a reduced resistance to drought, causing massive vegetation death. The internal surface of Kuye River Basin is unsustainable, with serious land desertification in a very fragile ecosystem. This causes the speed of improvement to have difficulty in restricting the speed of deterioration of the ecological environment quality. In addition, in rural areas with a high dependence on natural resources, the ecological environmental protection and economic and social welfare are complementary. Only by eliminating poverty can we effectively improve the environmental quality of the caves and wild rivers, to avoid achieving development at the expense of environment.

## 5. Conclusions

In this study, we used a PCA coupling NDVI, WET, NBDSI, and LST on the GEE platform to construct a macroscopic and efficient ecological and environmental quality monitoring method based on RSEI. We quantitatively analyzed the spatial and temporal distribution of the ecological and environmental quality in the Kuye River Basin. We obtained regional ecological environment quality information quickly providing an effective and simple means to reveal the dynamic changes of the ecological environment in the Kuye River Basin. Our analysis objectively and quantitatively investigated time and space and overcame the shortcomings of complex ecological evaluation indexes and cumbersome processes used in the past. The results show that the RSEI evaluation index can effectively reflect the ecological environment quality of the Kuye River Basin with high applicability. NDVI and WET had a positive effect on the ecological environment quality of the Kuye River Basin, while NDBSI and LST had a negative effect on the ecological environment quality. The inhibitory effect of NDBSI and LST on the ecological environment were significantly greater than the promoting effect of NDVI and WET on the ecological environment. The WET had a greater promoting effect on the ecological quality of the study area and had a better effect on the improvements in the ecological quality of the study area. The NDBSI index had a great inhibitory effect on the changes in ecological quality, indicating that urban expansion activities will lead to a significant reduction in the regional ecological quality.

Since 2000, the ecological environment quality of the Kuye River Basin has fluctuated and declined. Although a series of measures such as industrial structure adjustment, sand control, and grass planting have achieved initial results, reflecting the ecological environment has recovered to a certain degree. In some areas of the basin, however, the land desertification is serious, the vegetation is sparse, short and low coverage rate, and the ecosystem is fragile. The speed of improvement of ecological environment quality cannot catch up with the speed of deterioration of ecological environment quality. With the implementation of the basin development strategy, several energy and chemical bases have been built in the basin, the rapid expansion of cities and the rapid growth of population have rendered the basin’s ecological environment diverse, volatile, and fragile.

From the perspective of spatial and temporal distribution, the ecological and environmental quality of Kuye River Basin had a downward trend from 2000 to 2020. The overall ecological quality level was mainly moderate or poor. The mean value of the RSEI decreased from 0.595 in 2000 to 0.387 in 2020. The sum proportion of excellent RSEI grade areas decreased from 66.98% in 2000 to 3.2% in 2020. The sum proportion of medium, low, and poor RSEI grade areas increased from 33.02% in 2000 to 91.8% in 2020. The spatial distribution of RSEI decreased gradually from southeast to northwest. The degraded areas were mainly distributed in urban areas with frequent human activities. The excellent RSEI grade areas were mainly distributed in the eastern section of the watershed. The ecological environmental quality of the Yulin urban area and Shenmu County in the southern section of the watershed had a worsening trend compared with 2000.

To improve the ecological environment of the Kuye River Basin in the future, we should continue to strengthen the supervision of coal enterprises in the Kuye River Basin. All coal enterprises should deal with pollution at the source, and this should be combined with a series of industrial upgrading measures to improve the coal industry impacts on the environment. We will continue to strengthen the implementation of ecological remediation projects, strengthen the publicity and protection of the ecological environment, actively promote the transformation and development of mining areas, and strive to build a resource-saving and environmentally friendly development strategy. In the development planning of the Kuye River Basin, it is necessary to focus on green development by implementing scientifically supported projects returning farmland to forest or grassland. It is important to coordinate development with the ecological environment and land use if we are to achieve sustainable development in the basin.

## Figures and Tables

**Table 1:** First Principal Component (PC1) of Principal Component Analysis for the RSEI in 2000, 2005, 2010, 2015, and 2020.

PC1	2000	2005	2010	2015	2020
NDVI	0.081	0.084	0.081	0.086	0.091
WET	0.262	0.226	0.254	0.233	0.235
NDBSI	−0.851	−0.853	−0.861	−0.879	−0.891
LST	−0.351	−0.392	−0.357	−0.343	−0.365
Eigenvalue	0.267	0.243	0.273	0.262	0.351
Percentage of eigenvalue	85.61	86.17	86.83	85.95	90.01

**Table 2:** Statistical results of the various indicators and RSEI in each year.

Year	Indicators	NDVI	WET	NDBSI	LST	RSEI
2000	Maximum	0.729	0.194	0.377	24.481	1.000
	Minimum	−0.191	−0.541	−0.354	18.173	0.000
	Mean	0.428	0.106	0.282	19.521	0.595
	Standard deviation	0.122	0.047	0.129	3.255	0.134
2005	Maximum	0.782	0.234	0.458	26.923	1.000
	Minimum	−0.132	−0.743	−0.492	19.121	0.000
	Mean	0.431	0.014	0.321	20.139	0.525
	Standard deviation	0.201	0.001	0.013	4.127	0.147
2010	Maximum	0.773	0.219	0.698	24.694	1.000
	Minimum	−0.157	−0.636	−0.482	17.711	0.000
	Mean	0.336	0.021	0.332	20.333	0.483
	Standard deviation	0.122	0.001	0.125	2.999	0.138
2015	Maximum	0.813	0.286	0.744	25.878	1.000
	Minimum	−0.165	−0.672	−0.504	17.874	0.000
	Mean	0.277	0.036	0.423	20.536	0.429
	Standard deviation	0.098	0.001	0.228	2.867	0.159
2020	Maximum	0.774	0.205	0.799	26.756	1.000
	Minimum	−0.170	−0.914	−0.430	17.821	0.000
	Mean	0.017	0.051	0.528	20.602	0.387
	Standard deviation	0.001	0.007	0.186	2.345	0.112

**Table 3:** Area and percentage of each RSEI level in 2000, 2005, 2010, 2015, and 2020.

RSEI Level	2000		2005		2010		2015		2020	
	Area/km^2^	Pct./%	Area/km^2^	Pct./%	Area/km^2^	Pct./%	Area/km^2^	Pct./%	Area/km^2^	Pct./%
Poor	257.191	2.95	581.346	6.68	822.520	9.45	1010.791	11.61	3201.554	36.77
Fair	866.257	9.95	2690.249	30.90	2620.021	30.09	2605.081	29.92	3970.661	45.61
Moderate	1751.206	20.11	3041.527	34.94	2168.135	24.90	2265.580	26.02	820.006	9.42
Good	2989.513	34.34	1768.360	20.31	1817.897	20.88	2254.426	25.90	446.423	5.13
Excellent	2841.834	32.64	624.518	7.17	1277.428	14.67	570.122	6.55	267.356	3.07

## Data Availability

Not applicable.
